# Retinoprotective Effects of PACAP Eye Drops in Microbead-Induced Glaucoma Model in Rats

**DOI:** 10.3390/ijms22168825

**Published:** 2021-08-17

**Authors:** Edina Szabo, Evelin Patko, Alexandra Vaczy, Dorottya Molitor, Adrienne Csutak, Gabor Toth, Dora Reglodi, Tamas Atlasz

**Affiliations:** 1 Department of Anatomy, MTA-PTE PACAP Research Team, University of Pecs Medical School, 7624 Pecs, Hungary; szaboedina90@gmail.com (E.S.); evelin.patko@gmail.com (E.P.); vaczyalexandra@gmail.com (A.V.); molittty@gmail.com (D.M.); dora.reglodi@aok.pte.hu (D.R.); 2 Department of Ophthalmology, Clinical Centre, University of Pecs Medical School, 7632 Pecs, Hungary; csutak.adrienne@pte.hu; 3 Department of Medical Chemistry, Faculty of Medicine, University of Szeged, 6720 Szeged, Hungary; toth.gabor@med.u-szeged.hu; 4 Szentagothai Research Center, University of Pecs, 7624 Pecs, Hungary; 5 Department of Sportbiology, University of Pecs, 7624 Pecs, Hungary

**Keywords:** glaucoma, PACAP, eye drops, protection, intraocular pressure

## Abstract

Glaucoma is associated with increased intraocular pressure (IOP), causing the apoptosis of retinal ganglion cells (RGCs) and the loss of their axons leading to blindness. Pituitary adenylate cyclase activating polypeptide (PACAP) is neuroprotective in several neural injuries, including retinopathies. The aim of this study was to investigate the effects of PACAP1-38 eye drops in a model of glaucoma. IOP was elevated bilaterally by injections of microbeads to block the aqueous humor outflow. The control groups received the same volume of saline. Animals were treated with PACAP1-38 (1 µg/drop, 3 × 1 drop/day) or vehicle for 4 weeks starting one day after the injections. Retinal morphology by histology and optical coherence tomography, function by electroretinography, and IOP changes were analyzed. Animals were sacrificed 8 weeks after the injections. Microbeads injections induced a significant increase in the IOP, while PACAP1-38 treatment lowered it to normal levels (~10 mmHg). Significant retinal degeneration and functional impairment were observed in the microbead-injected group without PACAP1-38 treatment. In the microbeads + PACAP1-38 group, the retinal morphology and functionality were close to the normal values. In summary, our results show that PACAP1-38, given in form of eye drops, is neuroprotective in glaucoma, providing the basis for potential future therapeutic administration.

## 1. Introduction

Glaucoma refers to a group of optic neuropathies. The most common form of it is open-angle glaucoma, which is a progressive condition that develops by the blockage of the aqueous humor (AH) drainage system leading to intraocular hypertension. The increased intraocular pressure will cause the loss of the RGCs and their axons [1]. Today, treatments are limited to moderate the intraocular pressure (IOP) elevation; however, retinal degeneration continues to progress at a slower rate. There is an emerging need for therapeutic agents that can prevent apoptosis and exert a neuroprotective effect [2]. Although the exact underlying mechanism of RGC apoptosis in glaucoma has not been fully clarified [3], evidence shows that oxidative stress, glial activation, and inflammatory reactions play a role in the pathomechanism [4,5,6,7].

Pituitary adenylate cyclase activating polypeptide (PACAP) is an endogenous neuropeptide first isolated as a hypothalamic peptide in two biologically active forms (PACAP1-27 and PACAP1-38). It is the most conserved member of the secretin/glucagon/VIP family and it exerts diverse biological actions [8,9,10]. PACAP acts on G-protein coupled receptors, namely PAC1, VPAC1, and VPAC2 receptors [10,11]. Since its discovery, it has become evident that PACAP has strong neuroprotective effects in several in vivo and in vitro models such as Parkinson’s disease, cerebral ischemia, traumatic brain injury, and Huntington’s disease [12,13,14,15]. PACAP1-38 is now considered an effective neuroprotective and cytoprotective peptide with potential therapeutic effects. In the retina, PACAP has been shown to ameliorate lesions in several models of retinopathy. Our research team found that PACAP protects the ischemia-induced changes and promotes anti-apoptotic pathways [16,17]. Among others, PACAP has been shown to counteract the damaging effects of the excitotoxins glutamate and kainate, hyperoxia/hypoxia, oxidative stress, UV-light, hyperglycemia, optic nerve transection, and endotoxins [8,18,19,20].

Altogether, these findings give strong evidence that PACAP has potential therapeutic importance in severe retinopathy [21,22]. Previously, we have proven that PACAP, given in form of eye drops on the surface of the cornea, is able to pass through the ocular barriers to reach the retina with an appropriate vehicle and exert retinoprotective effects [23]. The topical administration would provide a non-invasive method for the treatment of ophthalmological diseases [24].

Therefore, the main purpose of the present study was to investigate the effects of PACAP1-38 eye drops in a rat model of hypertensive, primary open-angle glaucoma, using morphological, immunological, and functional techniques.

## 2. Results

### 2.1. Effect of PACAP Eye Drops on IOP

**Figure 1:**
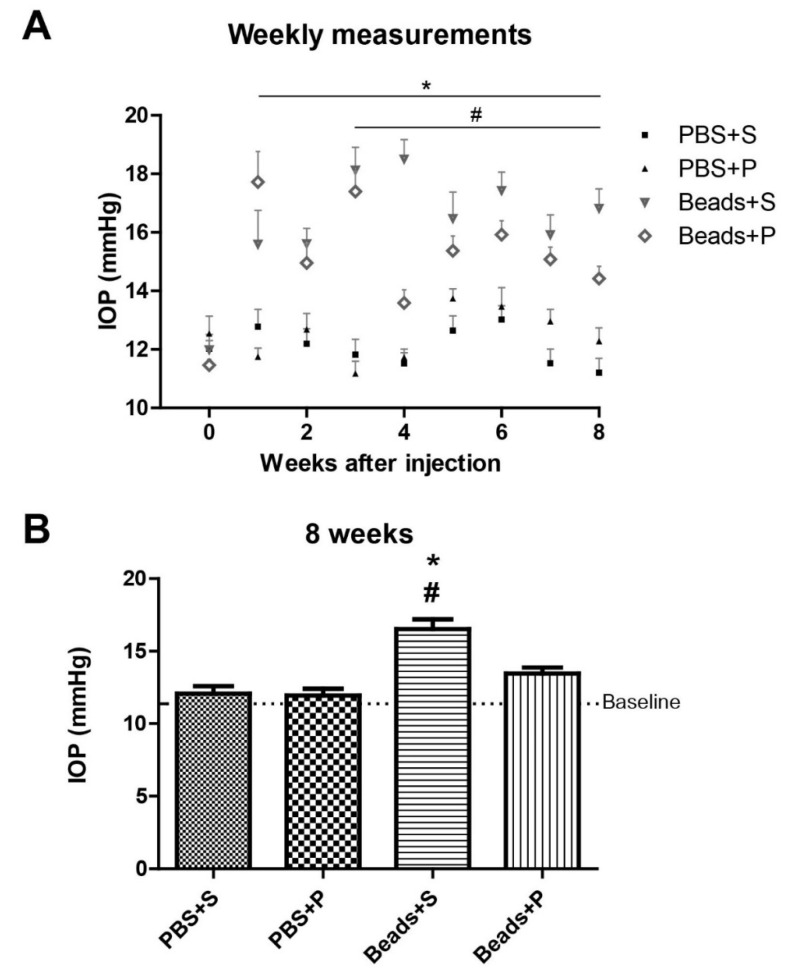
(**A**). IOP dynamics in the four examined groups (PBS + S, PBS + P, Beads + S, Beads + P) during the 8-week period. Intraocularly injected microbeads caused significant elevation of the IOP. (**B**). Bar chart shows the IOP in mmHg 8 weeks after the injection. Significant elevation of IOP was found in the Beads + S group compared to the control ones. PACAP1-38 eye drops resulted in the reduction of the IOP in the Beads + P group compared to the vehicle-injected group (Beads + S). Values are expressed in mean + SEM, analyzed by ANOVA and Bonferroni’s post hoc test. * Beads + S vs. PBS + S *p* < 0.05; # Beads + S vs. Beads + P *p* < 0.05. (Abbreviations: IOP: intraocular pressure; PBS: phosphate-buffered saline; S: Systane; P: PACAP1-38).

In control situations (phosphate-buffered saline (PBS) + Systane (S) and PBS + PACAP1-38 (P)), we did not detect any changes in the IOP (Figure 1A). In the Beads + S group, IOP increase developed during the observation period (Figure 1A), while topical PACAP1-38 administration attenuated the elevation of the IOP in the microbead-injected retinas. We already found more than 50% elevation of IOP in both groups receiving beads one week after the microbeads injection. On week 3, a significant difference (*p* < 0.05) started to develop between the two microbead-injected groups (Beads + S 55% IOP elevation; Beads + P 35% compared to PBS + S group). This tendency was observed during the 8 weeks, the percentages are the following: on week 4 Beads + S 53%, Beads + P 20%; on week 5 Beads + S 45%, Beads + P 19%; on week 6 Beads + S 33%, Beads + P 19%; on week 7 Beads + S 56%, Beads + P 36%; and on week 8 Beads + S 38%, Beads + P 12%.The differences were statistically significant starting from week 3, throughout the observation period. Detailed values of this statistically significant difference on week 8 is shown in Figure 1B. In the PBS-injected control groups (PBS + S and PBS + P), IOP levels remained close to the baseline on week 8 (PBS + S = 12.08 ± 0.5 mmHg; PBS + P = 11.95 ± 0.45 mmHg). In the microbead-injected vehicle-treated eyes (Beads + S), IOP showed a significant elevation (16.52 ± 0.68 mmHg) in contrast to the PACAP1-38-treated eyes (13.46 ± 0.42 mmHg; Beads + P).

### 2.2. Effects of PACAP1-38 Eye Drops Treatment on Histological Changes of the Retina

**Figure 2:**
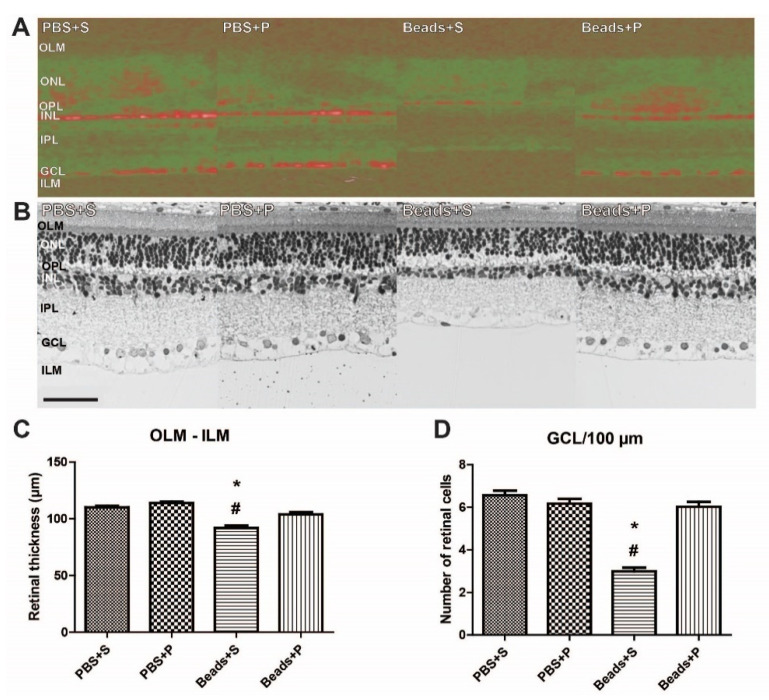
(**A**) Optical coherence tomography (OCT) shows the retinal layers in the four examined groups (PBS + S, PBS + P, Beads + S, Beads + P). In the OCT, retinal structure showed severe degeneration in the Beads + S group compared to the controls. A significant amelioration of the retinal structure was found after PACAP1-38 administration (Beads + P). (**B**) Representative light microphotographs of retinal sections in all groups. Retinal tissue from Beads + S group showed severe degeneration compared to PBS-injected retinas. The retained retinal structure following PACAP1-38 treatment (Beads + P) was similar to the control (PBS + P) retina. (**C**,**D**) Morphometric analysis of microbead-induced retinal damage. The degree of microbead-induced retinal neuronal degeneration and the neuroprotective effects of PACAP1-38 eye drops treatment were quantified by the cross-section of the retina from the outer limiting membrane to the inner limiting membrane (OLM–ILM), and the number of cells/100 µm ganglion cell layer (GCL) length. Values are expressed in mean ± SEM, analyzed by ANOVA and Fisher’s post hoc test. * Beads + S vs. PBS + S *p* < 0.05; # Beads + S vs. Beads + P *p* < 0.05. Bar: 50 µm. (Abbreviations: PBS: phosphate-buffered saline; S: Systane; P: PACAP1-38).

PACAP1-38 administration in PBS-injected animals did not result in any alterations in the retinal layers (Figure 2A–C). In vivo 3D OCT retinal images supported our histological findings (Figure 2A). Retinal layers in microbead-injected animals (Beads + S) showed signs of severe degeneration compared to the PBS controls (Figure 2A–D). A significant reduction was detected in the OLM–ILM thickness (91.81 ± 2.12 µm) in this group (Figure 2A–C). The number of cells in the GCL/100 µm was also significantly decreased (2.99 ± 0.18; Figure 2D). Topical administration of PACAP1-38 (Beads + P) led to significant protection in the retina. The microbead-injected PACAP1-38-treated retinas had a more preserved structure compared to the vehicle-treated retinas (Figure 2A,B) and resulted in a significantly better preserved whole retinal distance between the OLM–ILM (103.84 ± 2.02 µm). Quantitative morphometric analysis demonstrated that the loss in the number of cells in the GCL was also preserved (6.02 ± 0.23) in the PACAP1-38-treated groups (Figure 2D).

### 2.3. Effects of PACAP1-38 Treatment on Immunohistochemical Changes

**Figure 3:**
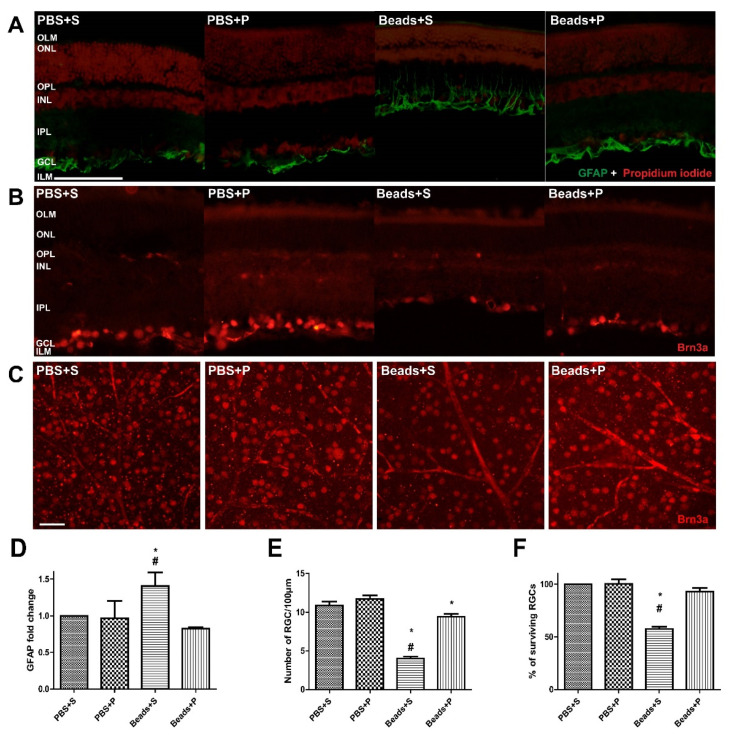
Representative vertical retinal sections (**A**,**B**) and whole-mount (retinal areas within 2 mm from the optic nerve) (**C**) stained by GFAP (**A**) and Brn3a (**B**,**C**) antibodies showing the effects of elevated IOP in the four examined groups (PBS + S, PBS + P, Beads + S, Beads + P). IOP resulted in massive elevation of GFAP immunopositivity (**D**), and reduction of Brn3a expression (**E**,**F**) in Beads + S group compared to the controls (PBS + S, PBS + P) and the PACAP1-38-treated (Beads + P) retinas. Statistical comparisons were made using ANOVA followed by Fischer’s (GFAP, Brn3a whole-mount) and Bonferroni’s (Brn3a section) post hoc analysis. Data are presented as means ± SEM. * Beads + S vs. PBS + S and Beads + P vs. PBS + P *p* < 0.05; # Beads + S vs. Beads + P *p* < 0.05. Bar: A, B: 50 µm; C: 100 µm. (Abbreviations: IOP: intraocular pressure; PBS: phosphate-buffered saline; S: Systane; P: PACAP1-38; GFAP: glial fibrillary acidic protein; Brn3a: brain-specific homeobox/POU domain protein 3A).

PBS-treated retinas did not show any remarkable immunofluorescent changes in either the vehicle-treated (PBS + S) or the PACAP1-38 eye drops (PBS + P) groups (Figure 3A–E). Significant glial fibrillary acidic protein (GFAP) upregulation was detected following microbeads injection in the retinas in the Beads + S group (Figure 3A,D). Expression was more intense in the inner retinal layers compared to the PACAP1-38-treated (Beads + P) retinas (Figure 3A,D). IOP resulted in massive loss of the Brn3a immunopositivity in RGCs (Beads + S) compared to the control eyes (PBS + S; Figure 3B,E). Glaucomatous retinas receiving PACAP1-38 eye drops (Beads + P) showed significantly smaller reduction in RGC cells (Figure 3A,D). To further confirm this quantitative observation, surviving RGCs were also counted in whole-mount retinas (Figure 3C,F). No significant differences were detected in PBS-injected groups (PBS + S and PBS + P). A reduced number of RGCs were observed in glaucomatous eyes (Beads + S) compared to the retinas in the PACAP1-38-treated group (Beads + P). We found that the decrease in Brn3a expression was counteracted by topical PACAP1-38 treatment.

### 2.4. Protective Effect of PACAP1-38 Eye Drops on Visual Responses after Ocular Hypertension

**Figure 4:**
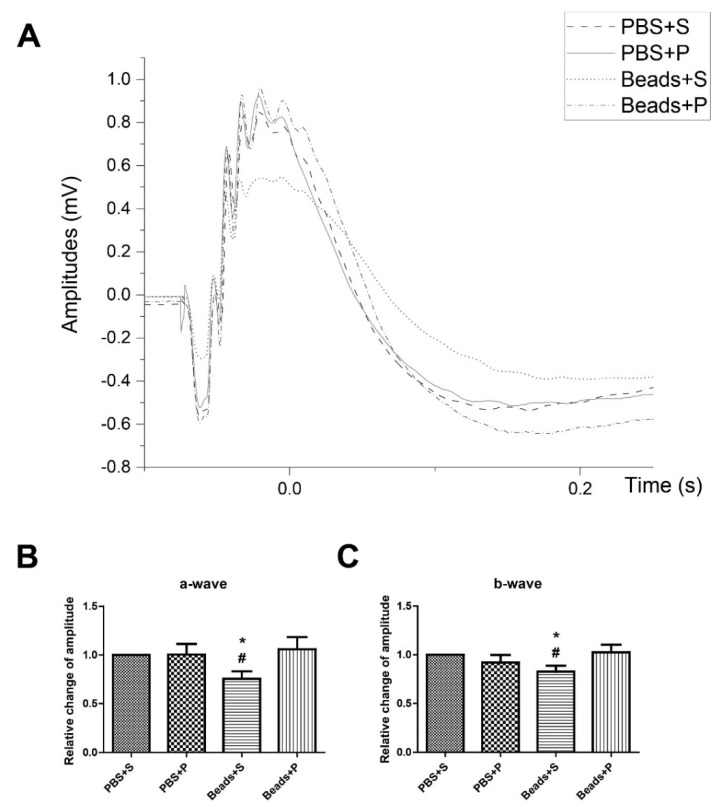
(**A**) Representative average ERG recordings of PBS + S, PBS + P, Beads + S, Beads + P groups. (**B**,**C**). Comparative analysis of the amplitude ratio of a-wave (**B**) and b-wave (**C**). ERG responses were similar in PBS + S and PBS + P rats under healthy conditions. Microbead-induced alterations (Beads + S) in the amplitudes of a- and b-waves compared to the control animals (PBS + S). ERG showed significant functional protection after PACAP1-38 eye drops treatment (Beads + P) in the microbead-injected eye compared to the Beads + S group. Data are given as mean ± SEM, analyzed by ANOVA and Fisher’s post hoc test. * Beads + S vs. PBS + S *p* < 0.05; # Beads + S vs. Beads + P *p* < 0.05. (Abbreviations: ERG: electroretinography; PBS: phosphate-buffered saline; S: Systane; P: PACAP1-38).

Representative ERG was recorded after 12 h dark adaptation (Figure 4). In control situations ERG waves were similar in the PBS + S and PBS + P retinas. In the glaucomatous vehicle-treated group (Beads + S), the light responses significantly decreased (Figure 4A). However, in the glaucomatous PACAP1-38-treated eyes (Beads + P), the waveforms were almost the same as in the PBS-injected groups. The scotopic a- and b-waves in the PBS-injected eyes were similar in the PBS + S (a-wave = 482.63 ± 31.81 µV; b-wave = 1331.60 ± 55.24 µV) and in the PBS + P (a-wave = 473.99 ± 50.03 µV; b-wave = 1195.77 ± 74.35 µV) ones (Figure 4B,C). We observed significant reduction of the a- and b-wave amplitudes in the Beads + S (a-wave = 347.89 ± 32.76 µV; b-wave = 1065.91 ± 67.6 µV) group compared to the PBS + S-treated animals. ERGs showed significant functional protection after PACAP1-38 administration (Beads + P) in the microbead-injected eye (Figure 4B,C).

## 3. Discussion

Glaucoma is a complex disease that is far from being completely understood. Animal models of glaucoma were developed more than twenty years ago, however, Urcola et al. was the first to apply injection of microbeads into the anterior chamber of rodent eyes to increase IOP [25]. Our model is based on Sappington’s earlier study using polystyrene microbeads to elevate the pressure [26]. In our present study, we could stably reproduce the retinal ganglion cell death induced by high IOP. Using this model, we proved that the neuropeptide PACAP was able to prevent the marked increase in the intraocular pressure and the significant ganglion cell loss.

PACAP has well-documented neuro- and general cytoprotective effects, including protective actions in several retinopathies. Among others, PACAP has been shown to reduce injuries in models of retinopathy of prematurity, diabetic retinopathy, and retinopathies induced by inflammation, ischemia, neurotoxicity, and UV light [8,17,19,21,22,23,27,28,29]. A difficulty of systemic treatment of retinopathies with PACAP is that the peptide has a short half-life in the serum due to the rapid degradation by the dipeptidyl-peptidase IV enzyme and that it is not known how it passes the blood–retina barrier. Most studies demonstrating the retinoprotective effects of PACAP, therefore, applied intravitreal treatment. However, intravitreal treatments have the disadvantage of being invasive, leading to secondary injures. We have previously shown that PACAP, given in form of eye drops, is able to pass the ocular barriers and reaches the retina in sufficient concentration to induce protective effects in a model of ischemic retinopathy [23]. The principal finding of our present study is that PACAP1-38, delivered as eye drops, has a protective role in microbead-induced glaucoma.

Similar to human glaucoma, the elevation of IOP in this rat model can lead to the loss of retinal ganglion cells. Normal IOP values were recorded around 10–12 mmHg, similar to those described by studies using Sprague–Dawley rats. In the glaucomatous eyes, beads with a diameter of 10 µm induced the blockage of the trabecular meshwork leading to IOP elevation. In our present study, we were able to reproduce this elevation in IOP after the injections and showed that treatment with PACAP1-38 eye drops could prevent this increase [30]. This was an unexpected positive finding of the present study. Although the exact mechanism is not known yet, the IOP-lowering effect of PACAP can be an additional protective factor in glaucoma. AH production and flow is a very tightly regulated process influenced by numerous factors and structures. It is not known yet how PACAP affects AH production and/or flow, but other cAMP-inducing substances have been described to have IOP-lowering effects. One of the possible mechanisms can occur through the cAMP level, as cAMP plays a critical role in the regulation of the AH production and outflow [31]. Moreover, PACAP can reduce the small GTPase RhoA which has a role in the regulation of trabecular meshwork [32,33].

Our histological findings are also in accordance with those of others [30,34,35]. Microbeads injections induced histological changes between the two limiting layers (OLM–ILM), similar to other retinal injures, such as LPS-induced retinal inflammation [19]. The degree of cell loss in our Beads + S group was similar to that reported in other studies using photocoagulation-induced glaucoma [36,37]. PACAP1-38 eye drops preserved the normal retinal structure and prevented the ganglion cell loss investigated by routine histology and the specific Brn3a immunohistochemical labeling. Müller glial cells are known to be over-activated in various injuries. Müller cells are known to have PAC1 receptor and PACAP can exert several effects on them [14]. Among others, PACAP has been shown to influence inflammatory cytokine (IL-6) expression in Müller cells [38,39,40]. The activation of Müller cells could be confirmed in our present study, demonstrating more intense GFAP labeling in hypertensive conditions, while in the PACAP-treated group, GFAP positivity was limited only to the end feet of the glial cells, in concordance with previous findings [19,23]. Seki et al., using another model for increased IOP with saline injection, have already demonstrated the protective effects of intravitreal injections of PACAP in the retina, focusing on the ganglion cell death. They suggested that PACAP1-38 can induce different signaling pathways depending on the concentration. Our findings support their results that PACAP1-38 has neuroprotective effects in hypertension-induced glaucoma. Our present results further confirm these earlier findings in a model more closely resembling the pathophysiological mechanisms of human glaucoma. In addition, we could show protective effects not only in the ganglion cell layer but also in other layers and in Müller cells, and could also confirm that the morphological improvement is associated with functional amelioration. Above all, we could provide evidence for the protective effects of PACAP in this model using a non-invasive eye drops application of PACAP1-38 [36].

To investigate whether the morphological amelioration by PACAP treatment is also reflected in functional improvement, we performed ERG measurements [41]. Scotopic ERG waveforms represent specific cell type activities. The activation of rods and cones results in the a-wave, while the activation of ON bipolar neurons, amacrine, and Müller glial cells forms the b-wave [42]. Here, we confirmed that PACAP treatment could also prevent the deterioration in visual function detected in the Beads + S group. This observation is in accordance with previous findings in ischemic retinopathy [41]. Although the two pathomechanisms differ, they also share some common features, as vascular dysregulation has also been described in glaucoma. We observed several functional alterations in the Beads + S group proving that low-to-moderate elevation of IOP is necessary to induce experimental glaucoma in rodents. In contrast, no such changes were observed in the PACAP-treated group.

Irreversible visual loss is a severe clinical issue commonly caused by glaucoma. Currently, there is no known effective neuroprotective therapy. Brain-derived neurotrophic factor (BDNF) injection into the vitreous body has proven to maintain the number of ganglion cells [43]. PACAP1-38 eye drops therapy has a similar potent preventive effect against retinal ganglion cell death in our glaucoma model. There is a need to develop an effective neuroprotection method which is able to interact with cellular signaling and promotes RGCs survival. The locally produced BDNF might be important in the RGC activation through the TrkB receptor [44]. The two factors are also linked to each other, as PACAP1-38 can induce the expression of BDNF via its specific PAC1 receptor and PACAP’s protective effects are partially mediated by BDNF in neuronal cells [45,46,47].

The microbead occlusion model of glaucoma represents an attractive model for determining the impact of PACAP on glaucoma. Our present findings further suggest that PACAP1-38 eye drops could be used in future therapeutic approaches. Taken together, PACAP1-38 eye drops treatment can provide a future accessory strategy designed parallel to the regular glaucoma treatments.

## 4. Materials and Methods

### 4.1. Animals

Adult male Sprague–Dawley (SD) rats (n = 50) weighing 300–500 g were used in this experiment. Animals were maintained under a 12 h light/dark cycle and fed and watered ad libitum. All the procedures were approved by the Animal Welfare Committee of the University of Pecs, and the National Scientific Ethical Committee on Animal Experimentation (ÁTET) at the Ministry of Agriculture, fully complied with the Decree No. 40/2013. (II. 14.) of the Hungarian Government and the EU Directive 2010/63/EU on the protection of animals used for scientific purposes (ethical permission numbers: BA02/2000-16/2017, and ARVO Statement for the Use of Animals in Ophthalmic and Vision Research. Rats were divided randomly into four experimental groups: (i) PBS + vehicle (Systane (S)) n = 8; (ii) PBS + PACAP1-38 (P) n = 8; (iii) microbeads + vehicle (S) n = 17; and (iv) microbeads + PACAP1-38 n = 17, referred to as PBS + S; PBS + P; Beads + S; and Beads + P, respectively.

### 4.2. Administration of Microbeads

The injection of microbeads was performed as previously described by Sappington et al. [26] to induce high IOP in rats. Animals were anesthetized with intraperitoneal ketamine (90 mg/kg; Calypsol, Richter Gedeon, Hungary) and xylazine (10 mg/kg; Sedaxylan, Dechra, Netherlands) injection. Before the microbeads injection, we applied the disinfectant Braunol solution (B. Braun Medical AG, Switzerland) to prevent infections. Fluorescent (580/603 nm) polystyrene microbeads (FluoSpheres™ Polystyrene Microspheres; 10 µm Thermo Fisher Scientific; Waltham, MA, USA) (3.6 × 10^6^ beads/mL; 10 µL/injection) were injected into the anterior chamber (AC) of both eyes by Hamilton syringe (33G needle). After administration of microbeads, anti-inflammatory eye drops (Tobrex, 3 mg/mL; Alcon, Budapest, Hungary) were used to prevent inflammation and promote corneal healing. In the control groups, eyes received an injection with the same volume (10 µL) of PBS. Two weeks after the injection, we repeated the same procedure.

### 4.3. IOP Measurement

One day before the intervention, IOP was measured in both eyes with a rebound tonometer (Tonolab, Icare; Vantaa, Finland). To avoid IOP fluctuation [48,49] due to the circadian cycle, we measured IOP at the same time of the day throughout the 8 weeks (10–11 a.m., once a week). For each eye, the mean value of three consecutive measurements was used. After IOP measurement, lubricant ointment was also applied to the ocular surface.

### 4.4. Eye Drops Treatment

After the microbeads injection, the eyes were treated with Systane solution (S) (Alcon, Budapest, Hungary) or PACAP1-38 (P) eye drops (1 µg/drop) (PACAP1-38 was synthesized at the Department of Medical Chemistry, University of Szeged, Szeged, Hungary), according to previous descriptions [50]. Systane solution was used as a vehicle. Rats were treated 3 times a day with 1 drop PACAP1-38 solution, for 4 consecutive weeks.

### 4.5. Optical Coherence Tomography Examination

Noninvasive, in vivo imaging was performed with Optical Coherence Tomography (OCT) (Bioptigen, Morrisville, NC, USA). This technique gives the chance to obtain high-resolution images of the anterior chamber or the retina in real-time. OCT imaging was performed 1 day before the microbeads injections. Anesthesia was carried out by intraperitoneal injection of ketamine (90 mg/kg) and xylazine (10 mg/kg). Animals with ocular inflammation were excluded from further experiments and were not included in the final number of the animals. The pupils were dilated using eye drops that contained 0.01% atropine. During the procedure, we applied artificial tear to protect the corneal surface (Systane solution (Alcon, Budapest, Hungary). Images of the retina were collected before and 8 weeks after the injections.

### 4.6. Morphological and Morphometric Analysis

Rats (n = 16) were killed 8 weeks after the microbeads injections. The eyes were removed and dissected in 0.1 M PBS and fixed in 4% paraformaldehyde dissolved in 0.1 M phosphate buffer (PB). After fixation, eyecups were washed in 0.1 M PBS for one hour, then our samples were dehydrated in ascending row of alcohol. These samples were embedded in Durcupan ACM resin (Sigma-Aldrich, Budapest, Hungary) and placed into thermostat at 56 °C for 72 h. From our histological blocks, semi-thin sections (2 µm) were made by microtome (Reichert Ultracut E, Wien, Austria) and were stained with routine histological staining (1% toluidine blue solution, Sigma-Aldrich, Budapest, Hungary). Microphotographs were made from our sections and were analyzed with light microscopy (Nikon Eclipse 80i). Measurements were taken from the digital photographs with the Nikon Nis-Elements program. Four tissue blocks from animals were made and central retinal areas within 2 mm from the optic nerve were used for the measurements. The following parameters were analyzed: (i) retinal cross-section between the outer and inner limiting membranes (OLM–ILM), as well as the (ii) number of cells/100 μm section length in the ganglion cell layer (GCL).

### 4.7. Immunohistochemistry

For immunohistochemistry, retinas (n = 32) were dissected in 0.1 M PBS and fixed in 4% paraformaldehyde dissolved in 0.1 M phosphate buffer (PB) for 2 h at room temperature followed by washing 0.1 M PBS for one hour. Then, eyecups were immersed into a 10–20–30% sucrose solution and embedded in optimal cutting temperature compound mounting media (Tissue Freezing Medium, Mount Waverley, Australia). Next, 15–17 μm thin sections were cut (central retinal areas within 2 mm from the optic nerve) on gelatin-coated slides with cryostat (Leica CM1950, BioMarker, Budapest, Hungary) and they were processed for further immunohistochemical analysis.

After rehydration with 0.1 M PBS, sections were blocked in 5% normal donkey serum, 3% bovine serum in PBS, and 0.3% Triton™ X-100 (PBST) at room temperature for 2 h to minimize nonspecific labeling. Sections were incubated overnight at 4 °C with primary polyclonal antibodies: (i) mouse anti-Brn3a (brain-specific homeobox/POU domain protein 3A) (Sigma-Aldrich, Budapest, Hungary), and (ii) rabbit anti-GFAP (Sigma-Aldrich, Budapest, Hungary) diluted in 1:200 in antibody diluting buffer. Immunoreactivity was detected with Alexa Fluor-594, donkey anti-mouse (Jackson ImmunoResearch, Cambridgeshire, UK) and Alexa Fluor-488, donkey anti-rabbit (Jackson ImmunoResearch, Cambridgeshire, UK) diluted 1:400 in PBST.

After the secondary antibody, sections were washed in 0.1 M PBS for one hour. Cell nuclei were stained with propidium iodide (PI) (Sigma-Aldrich, Budapest, Hungary). For control experiments, primary antibodies were omitted, resulting in no specific staining. After washes, slides were coverslipped with Fluoroshield (Sigma-Aldrich, Budapest, Hungary). Microphotographs were made by Nikon Eclipse 80i fluorescence microscope. Photographs were further processed with the Adobe Photoshop CS6 program (Adobe Systems, Inc., San Jose, CA, USA). Images were adjusted for contrast only; they were aligned, arranged, and labeled using the functions of the Photoshop CS6 program. Fold change of GFAP-positive area was measured by ImageJ software (National Institutes of Health, Bethesda, MD, USA). We quantified the RGC number with the previously described method (morphometric analysis).

### 4.8. Retinal Whole Mounts

Eyes (n = 36) were dissected in 0.1 M PBS and fixed in 4% paraformaldehyde dissolved in 0.1 M PB for 2 h at room temperature followed by washing 0.1 M PBS for one hour. After washing, retinas were removed and four small cuts were made. Retinas were placed in a 24 well-plate and were blocked in 5% normal donkey serum, 3% bovine serum in 0.3% PBST for 1 h. Primary mouse anti-Brn3a (Sigma-Aldrich, Budapest, Hungary) was diluted in PBST and incubated overnight at 4 °C. Immunoreactivity was visualized with Alexa Fluor-594 donkey anti-mouse (Jackson ImmunoResearch, Cambridgeshire, UK) diluted 1:400 in PBST. After, the secondary antibody samples were washed in 0.1 M PBS for one hour. Slides were mounted with Fluoroshield (Sigma-Aldrich, Budapest, Hungary) mounting medium. Brn3a-positive RGCs were counted in 4 regions (one region per retinal quadrant from the same area as previously described) each of area 50,000 µm^2^. Counting was managed by ImageJ (National Institutes of Health, Bethesda, MD, USA). Images were analyzed with Nikon Eclipse 80i epifluorescence microscope. Photographs were also further processed with the Adobe Photoshop CS6 program (Adobe Systems, Inc., San Jose, CA, USA). Images were adjusted for contrast only; they were aligned, arranged, and labeled using the functions of the Photoshop CS6 program.

### 4.9. Electroretinography

Scotopic electroretinography (ERG) responses were recorded from both eyes 1 day before and 8 weeks after microbeads injection. Before the measurement, animals were dark-adapted overnight (>12 h) and all set-up preparations were performed under dim red light (632 nm). For the examination, animals were anesthetized with an intraperitoneal injection of ketamine (90 mg/kg and xylazine (10 mg/kg) [51]. Pupils were dilated with one drop of 0.01% atropine before the ERG recording. Rats were placed on a heating pad and ERGs were recorded by active electrodes from the corneal surface and the reference electrodes were placed subcutaneously on the head. Ground electrode was also used subcutaneously under the skin of the back. The light pulses intensity (5cd s/m^2^, 0.25 Hz, 503 nm green LED light) was pre-amplified, amplified (2.000×, Bioamp SbA4-V6, Supertech, Hungary), and recorded with an A/D converter (Ratsoft-Solar Electronic). Responses (n = 50/eye) were averaged with Ratsoft software [41]. The following parameters were measured: amplitude of the a-wave (from baseline to the trough of the a-wave) and the amplitude of the b-wave (from the trough of the a-wave to the peak of the b-wave). The recording dataset was averaged and then further processed to examine the waveform of the electroretinograms with Origin Pro 2018 (Macasoft, Hungary).

### 4.10. Statistical Analysis

Statistical comparisons were made using the two-way ANOVA followed by Fischer’s (histology; ERG; GFAP; Brn3a whole-mount) and Bonferroni’s (IOP; Brn3a section) post hoc analysis. Data are presented as means ± SEM. Differences with *p* < 0.05 were considered significant.

## 5. Limitations

The limitation of this model in rats is that there is no clear relationship between the volume/size of injected microbead and the IOP elevation [52]. Shappington et al. (2010) evaluated the effect of different volumes on the IOP elevation. According to their results, after single injection of microbeads (5 µL or above) IOP increased for 2 weeks. It extended until 8 weeks with repeated injections in rats [26]. Urcola et al. (2006) used the microbead injection with hydromethylcellulose to achieve a higher IOP level [25]. One of the inconveniences is to keep the microbeads in the anterior chamber. Compared to human eyes, in rodents, it is more difficult to reach a self-sealing corneal wound after bead injection due to the thin cornea.

## Data Availability

The dataset generated during this study is available from the corresponding author upon reasonable request.
